# PEA-15 engages in allosteric interactions using a common scaffold in a phosphorylation-dependent manner

**DOI:** 10.1038/s41598-021-04099-6

**Published:** 2022-01-07

**Authors:** Joyce Ikedife, Jianlin He, Yufeng Wei

**Affiliations:** 1 Department of Chemistry, New Jersey City University, Jersey City, NJ 07305 USA; 2 Ministry of Natural Resources, Third Institute of Oceanography, Xiamen, 361005 Fujian China

**Keywords:** Molecular modelling, Phosphoproteins

## Abstract

Phosphoprotein enriched in astrocytes, 15 kDa (PEA-15) is a death-effector domain (DED) containing protein involved in regulating mitogen-activated protein kinase and apoptosis pathways. In this molecular dynamics study, we examined how phosphorylation of the PEA-15 C-terminal tail residues, Ser-104 and Ser-116, allosterically mediates conformational changes of the DED and alters the binding specificity from extracellular-regulated kinase (ERK) to Fas-associated death domain (FADD) protein. We delineated that the binding interfaces between the unphosphorylated PEA-15 and ERK2 and between the doubly phosphorylated PEA-15 and FADD are similarly composed of a scaffold that includes both the DED and the C-terminal tail residues of PEA-15. While the unphosphorylated serine residues do not directly interact with ERK2, the phosphorylated Ser-116 engages in strong electrostatic interactions with arginine residues on FADD DED. Upon PEA-15 binding, FADD repositions its death domain (DD) relative to the DED, an essential conformational change to allow the death-inducing signaling complex (DISC) assembly.

## Introduction

Phosphorylation is arguably the most prevalent posttranslational modification that could completely alter the fate of the cell. Phosphorylation controls and regulates cell cycle progression^1^ and cell proliferation^2^, and plays critical roles in T-cell signaling and immune responses^3^ and T cell proliferation and survival^4^. As we proposed earlier, a delicate balance of phosphorylation levels inside cells must be vigorously maintained to keep the cells at optimal conditions, which we have termed phosphorylation homeostasis^5^. Here we report our molecular dynamics (MD) studies on a small, non-catalytic protein, phosphoprotein enriched in astrocytes, 15 kDa (PEA-15), which displays significant conformational changes allosterically mediated by its phosphorylation states. Phosphorylation of PEA-15 completely switches its binding specificity and the related regulatory pathways and potentially determines the cell fate.

First identified in astrocytes as a substrate for protein kinase C (PKC)^6^, PEA-15 is ubiquitously expressed in all types of cells and tissues and is highly conserved among mammals^7^. PEA-15 has no catalytic activity, but it plays a significant role in regulating both cell proliferation and apoptosis (programmed cell death) through engaging in protein–protein interactions (PPIs) with either the mitogen-activated protein (MAP) kinases, extracellular signal-regulated kinases (ERK) 1 and 2, or Fas-associated death domain (FADD) protein. Structurally, PEA-15 consists of a canonical six-helix bundled death-effector domain (DED) at the N-terminus, and a long, irregularly structured C-terminal tail^8,9^. While the C-terminal tail is intrinsically disordered, it is evident that the C-terminal tail is crucial in ERK binding^8,10,11^, and contains two phosphorylation sites, Ser-104, the substrate for PKC^6,12^, and Ser-116, the substrate for protein kinase B/Akt^13^ and Ca^2+^-calmodulin dependent protein kinase II (CaMKII)^12^. Unphosphorylated PEA-15 interacts with ERK1/2, blocking ERK-dependent transcription and proliferation through the mediation of nuclear transport by the PEA-15 nuclear exporting sequence (NES) that prevents the entry of ERK1/2 into the nucleus^14,15^. Double phosphorylation of both serine residues (*p*-S104/*p*-S116) alters the binding specificity of PEA-15 from ERK1/2 to FADD, preventing the recruitment and activation of procaspase-8 at the death-inducing signaling complex (DISC), and blocking death receptor-initiated apoptosis^16–18^. Specifically, *p*-S104 blocks the interaction with ERK, and *p*-S116 promotes the recruitment of PEA-15 to the DISC in vivo^19^. In other words, unphosphorylated PEA-15 is a tumor suppressor as it inhibits ERK-dependent proliferation, while the doubly phosphorylated protein becomes a tumor promotor as it blocks apoptosis^20^. PEA-15 phosphorylation fits very well into the hallmark of almost all tumors in that it promotes unrestricted proliferation and inhibits apoptosis^5^. However, it is still unclear how phosphorylation at an intrinsically disordered region of PEA-15 could completely switch the binding specificity from one protein to another.

To answer these questions, we conducted molecular dynamics (MD) simulations on the free PEA-15, the doubly phosphorylated *p*-S104/*p*-S116 PEA-15 (PEA-15pp), and the complexes between unphosphorylated PEA-15 and ERK2, and between PEA-15pp and FADD. Here we report a conformational control mechanism mediated by the phosphorylation on the C-terminal tail, and a common scaffold on PEA-15 in the interactions with both ERK2 and FADD. The binding specificity is allosterically controlled by electrostatic interactions involving phosphorylated Ser-116. We also report, for the first time, a DED complex model formed between PEA-15pp and FADD, and highlight a critical conformational reorientation between the two domains of FADD. This DED complex model and the reorientation in FADD provide significant insights into the formation of the DISC and the activation of the extracellular receptor-initiated apoptosis pathway.

## Results

### Structures and conformations of unphosphorylated and phosphorylated PEA-15

For unphosphorylated PEA-15 and phosphorylated PEA-15pp proteins in their free forms, 50-ns MD simulations were performed. The root-mean-square deviation (RMSD) plots for both PEA-15 and PEA-15pp simulations indicated that the structures reached equilibrium after 30 ns. The large fluctuations in RMSD for the full-length proteins are mostly due to the intrinsically disordered nature of the C-terminal tail, as the DED in both models displays quite moderate RMSD (Supplementary Fig. S1). The subsequent analyses were performed using the last 20 ns of the simulations for both PEA-15 and PEA-15pp. The disordered nature of the C-terminal tail in both unphosphorylated and phosphorylated free-form proteins is clearly visible from the per-residue root-mean-square fluctuation (RMSF) analyses (Supplementary Fig. S2), with RMSF values for the C-terminal tail much higher than those of the DED, indicating high flexibility and elevated motion of the disordered tail.

**Figure 1:**
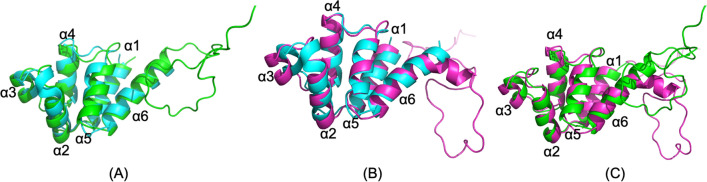
Superimpositions of (**A**) simulated free PEA-15, unphosphorylated (green) and NMR structure 2LS7 (cyan), (**B**) simulated free PEA-15pp, doubly phosphorylated p-S104, p-S116 (magenta) and NMR structure 2LS7 (cyan), and (**C**) simulated free PEA-15, unphosphorylated (green) and simulated PEA-15pp, doubly phosphorylated (magenta). Note that NMR structure 2LS7 contains only DED structure without the C-terminal tail. The simulated structure of unphosphorylated PEA-15 closely resembles the experimental NMR structure for the DED (RMSD 1.499 Å), while the simulated phosphorylated PEA-15pp has a very different conformation from the NMR structure (RMSD 2.027 Å). The simulated unphosphorylated PEA-15 and phosphorylated PEA-15pp also display conformational differences, with an RMSD of 1.994 Å. The molecular structures were visualized and generated using PyMOL version 2.4 (http://www.pymol.org/).

The simulated structure of the unphosphorylated PEA-15 closely resembles the experimental NMR structure (PDB ID 2LS7), with the backbone RMSD between the simulated and experimental DED structures of 1.499 Å (Fig. 1A). Although the simulated PEA-15pp structure largely maintains a six-helix bundle fold, the phosphorylation at the C-terminal serine residues induces some important changes from the unphosphorylated state (Fig. 1B). Most remarkably, the α3 helix shifts considerably from the position in the unphosphorylated structure, and the α6 helix exhibits a kink at residue Arg-85 in the phosphorylated structure. The backbone RMSD of the DED residues between PEA-15pp and experimental unphosphorylated structure is 2.027. The overlay of simulated PEA-15 and PEA-15pp structures shows the same distinction, with backbone RMSD of DED residues of 1.994 Å (Fig. 1C). The conformational change in PEA-15pp DED is allosterically induced by double phosphorylation at the C-terminal tail and is mediated by surface polar interactions, including hydrogen bonding and electrostatic interactions among polar and charged residues on DED (detailed discussions below).

### The complex structure and the binding interface between unphosphorylated PEA-15 and ERK2

For the two complexes formed between unphosphorylated PEA-15 with ERK2 (PEA-15/ERK2) and between phosphorylated PEA-15pp with FADD (PEA-15pp/FADD), 150-ns MD simulations were performed. The simulations for the two complexes stabilized after 100 ns (Supplementary Fig. S3). The analyses for these two complexes were conducted using the last 50-ns trajectories. In these complexes, it is noticeable from the RMSF plots that the PEA-15 protein greatly reduces the motion and flexibility of the C-terminal tail compared to the free-form proteins due to strong interactions between the tail and the interacting proteins (Supplementary Fig. S4). In the PEA-15/ERK2 complex, tail residues 121–129 tightly bind to ERK D-peptide recruitment site (DRS, also termed as DEJL, or docking site for ERK and JNK, LXL or kinase interaction motif), significantly immobilizing the C-terminal tail^11,21^. In the PEA-15pp/FADD complex, the phosphorylated *p*-S116 residue interacts strongly with R34 and R38 on FADD (see below), stabilizing the tail in the complex.

**Figure 2:**
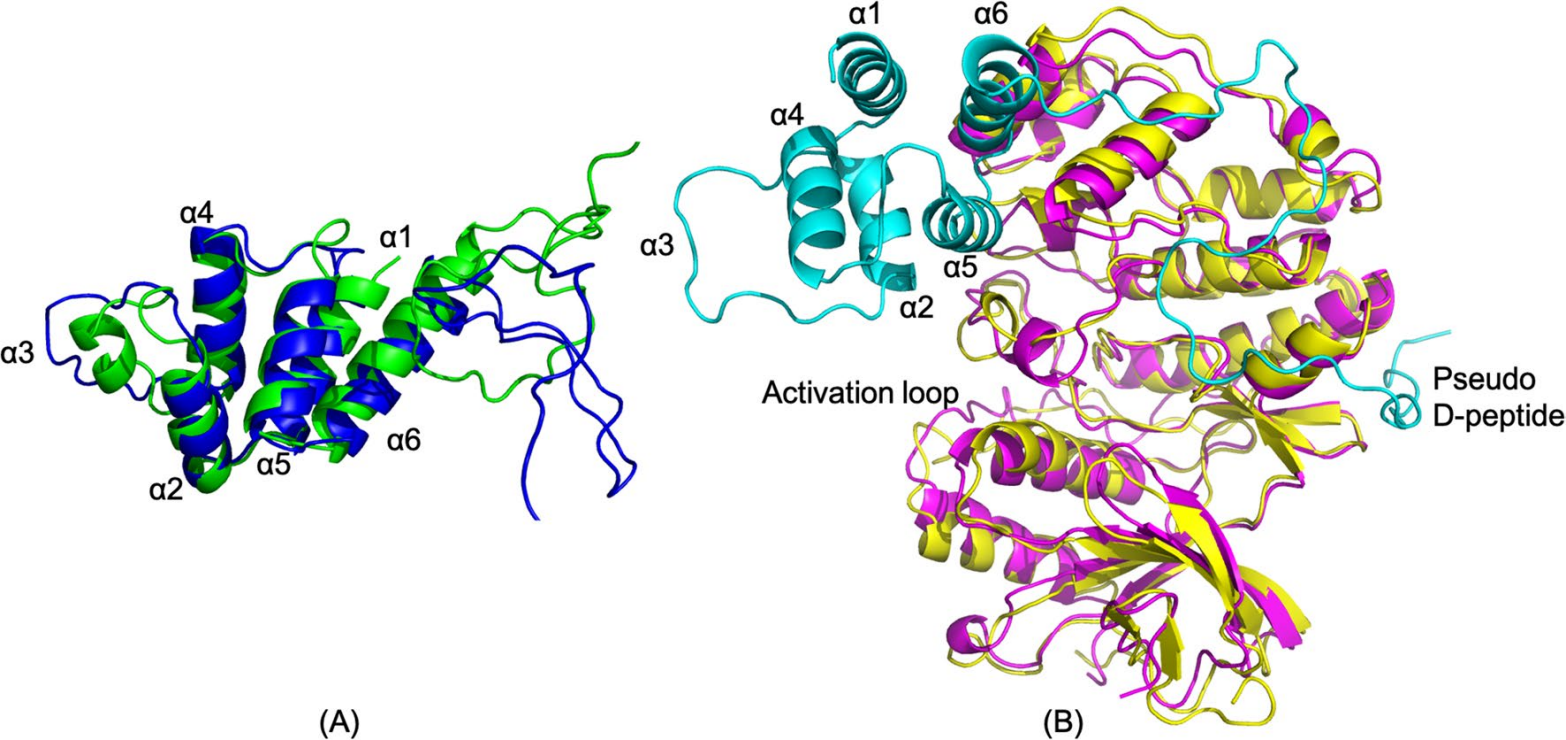
The simulated complex structure of PEA-15/ERK2. (**A**) PEA-15 in the complex (green) is superimposed with its free form (blue) with the RMSD 2.734 Å for the DED. Helix α3 is uncoiled and shifts in α2 and α4 can also be observed. (**B**) ERK2 in the complex is superimposed with the crystal structure of 4IZ5 with the RMSD 2.002 Å. The overall backbone of ERK2 does not show any significant difference between the simulated and crystal structure. The molecular structures were visualized and generated using PyMOL version 2.4 (http://www.pymol.org/).

In the PEA-15/ERK2 complex structure (Fig. 2A), PEA-15 exhibits a large conformational change in the DED, particularly at helix α3, which is completely uncoiled. Helices α2 and α4 also display slight shifts from their free-form positions. Helices α1, α5, and α6 have mostly maintained their positions. The backbone RMSD of PEA-15 DED between the complex and the free-form is 2.734 Å. The unwinding of the α3 helix is consistent with previous experimental results, including our NMR dynamics study, which indicated increased dynamics of α3 residues^11^, the X-ray crystallography study of PEA-15/ERK2 complex, which showed no electron density in this region^21^, and our CS-Rosetta model of PEA-15 in the complex, which characterized helix α3 as disordered^22^.

The ERK2 structure in the simulated PEA-15/ERK2 complex does not exhibit much difference from the initial structure derived from crystal structure 4IZ5, with a backbone RMSD of 2.002 Å (Fig. 2B). Most of the variations are localized in the loop regions, while the regular secondary structural components remain mostly intact, indicating a relatively rigid ERK2 in the complex. The per-residue RMSF is relatively small (< 0.3 Å) throughout the ERK2 sequence, except for the activation loop preceding the phosphorylation sites of Thr185 and Tyr187, and a long loop near the end of the C-lobe of ERK2 (Supplementary Fig. S5A).

**Figure 3:**
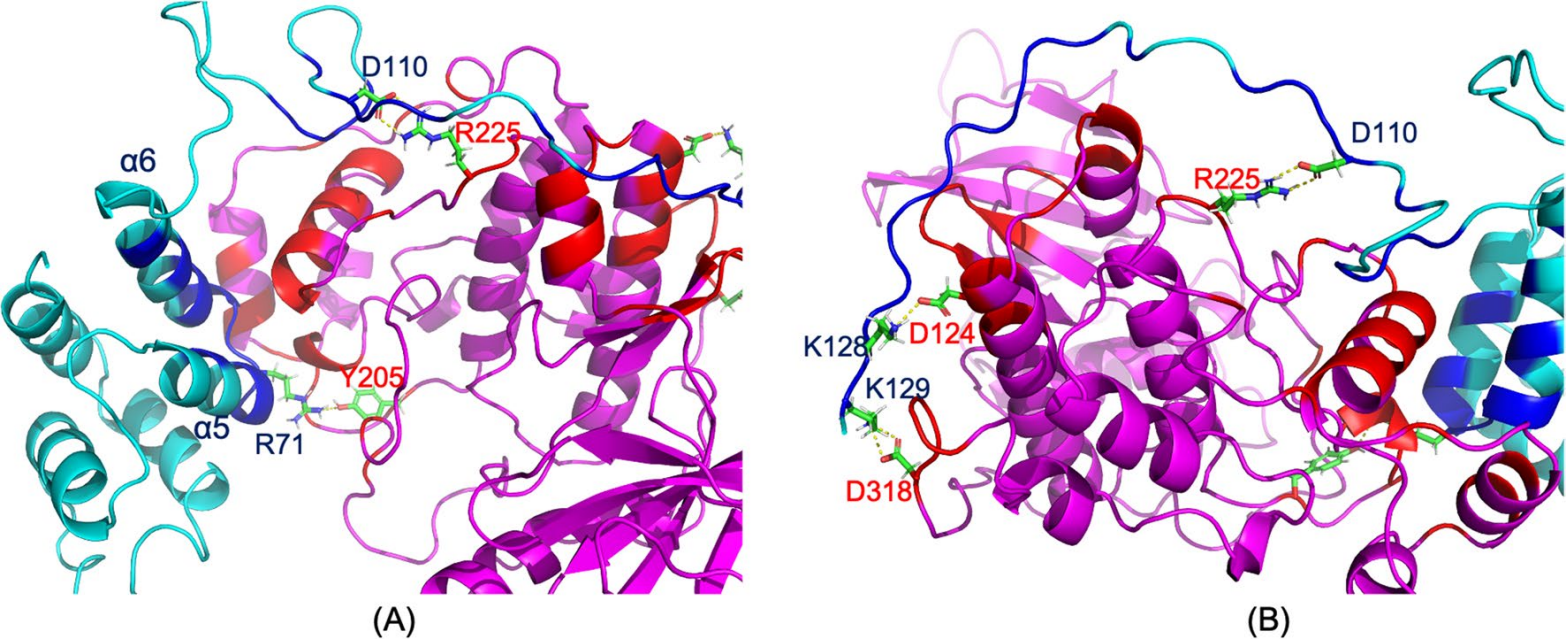
Binding interfaces in the simulated PEA-15/ERK2 complex. PEA-15 is shown in cyan and ERK2 in magenta. Interface residues on PEA-15 are colored in dark blue, and residues on ERK2 are colored in red. (**A**) The interface between PEA-15 DED and ERK2 DEF docking site. PEA-15 interacts with ERK2 with its helices α5/α6, including direct interaction between PEA-15 R71 and ERK2 Y205, consistent with crystal structure 4IZ5. (**B**) Interface between PEA-15 C-terminal tail and ERK2 D-peptide binding site, including interactions between PEA-15 K128 and ERK2 D124 and PEA-15 K129 and ERK2 D318. Another interaction that can be observed is between PEA-15 D110 and ERK2 R225. There are no direct interactions between either phosphorylation sites, S104 and S116, and ERK2 residues. The molecular structures were visualized and generated using PyMOL version 2.4 (http://www.pymol.org/).

The two binding interfaces between PEA-15 and ERK2 revealed in this MD study are consistent with earlier crystal structures^21^. PEA-15 utilizes its helices α5 and α6 of the DED to interact with the docking site for ERK, FxF (DEF) of ERK2, and engages its C-terminal tail to interact with the DRS of ERK2. Arg-71 on the loop between helices α5 and α6 of PEA-15 DED interacts directly to Tyr-205 of ERK2 DEF (Fig. 3A), while the charge-triad residues (D19, R72, and D74) of PEA-15 are not directly involved in binding to ERK2. PEA-15 C-terminal residues 121–129 interact with the ERK2 DRS residues as it contains a reversed pseudo-d-peptide sequence (Fig. 3B)^23,24^. Particularly, Lys-128 on the PEA-15 C-terminus directly interacts with Asp-124 of ERK DRS. The C-terminal residues 101–110 also exhibit significant interactions with ERK2, including the interaction between Asp-110 of PEA-15 and Arg-225 of ERK2 (Fig. 3B). The atomic distances of these intermolecular interactions are plotted in Supplementary Fig. S6A. All distances seemed to be stabilized after 120 ns of simulation. It is worth noting that the two phosphorylation sites, Ser-104 and Ser-116, of PEA-15 are not directly involved in the interaction to ERK2, which may explain the observation that phosphorylation states of PEA-15 do not affect the binding affinity with ERK2 in vitro^24^. These interactions can also be observed in the pairwise mean-smallest-distance maps. In the intermolecular regions of the PEA-15/ERK2 map (Supplementary Fig. S7A), short distances are identifiable in both the α5/α6—DEF and the C-terminal tail—DRS regions.

### The complex structure and the binding interface between phosphorylated PEA-15pp and FADD

**Figure 4:**
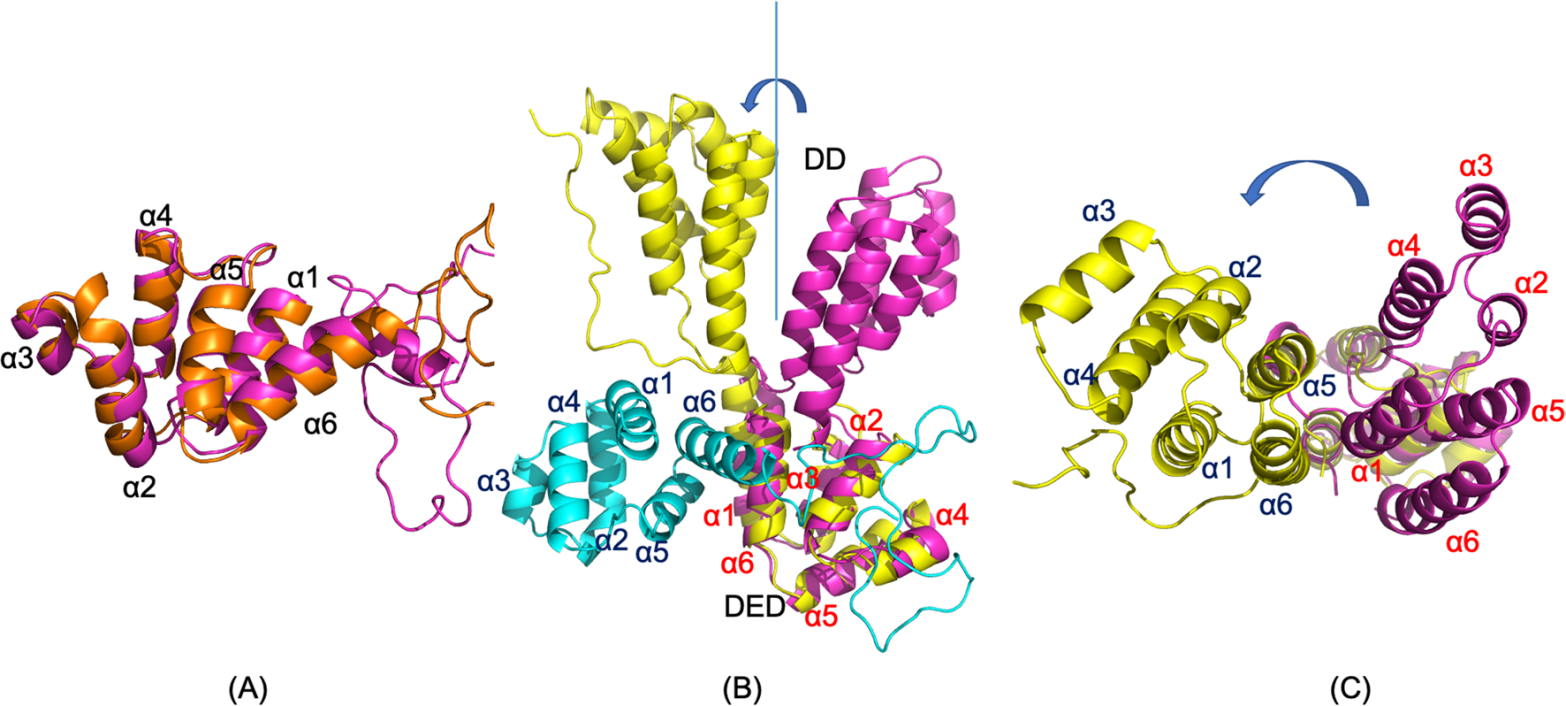
The simulated complex structure of PEA-15pp/FADD. (**A**) PEA-15pp in the complex (orange) is superimposed with free-form PEA-15pp (magenta) with the RMSD of 0.803 Å before the kink on helix α6. There is no significant conformational change between the free and bound forms. (**B**) FADD in the complex (yellow) is superimposed with the NMR structure of intact FADD, 2GF5 (magenta), over DED residues (RMSD 2.118 Å). PEA-15pp in the complex is colored cyan. DED–DED interaction in the complex is orthogonal in directions. The FADD DD in the complex rotated about 90° from the free form around the linker between the two domains. (**C**) same as (**B**) but observed from the top of FADD DD to illustrate the relative positions of the six helices on DD. PEA-15pp is not shown for clarity. The molecular structures were visualized and generated using PyMOL version 2.4 (http://www.pymol.org/).

FADD is comprised of an N-terminal death effector domain (DED) and a C-terminal death domain (DD), followed by a short tail. It is the adapter protein between the DD-containing death receptor, Fas, and the DED-containing procaspase-8, promoting the formation of the DISC through specific homotypic domain–domain interactions^25^. In the PEA-15pp/FADD complex structure, the phosphorylated PEA-15pp does not undergo an additional conformational change in the DED from the free-form structure, and the kink at the end of the helix α6 unwinds to become unstructured (Fig. 4A). The backbone RMSD for PEA-15pp DED (before the kink) is only 0.803 Å between the free and FADD-bound structures. The simulation results of PEA-15pp in its free and FADD-bound form are consistent with our initial NMR data, which indicated that phosphorylation at S104 and S116, mimicked by serine to aspartic acid mutation, on the C-terminal tail stimulates a conformational change at the DED, and FADD binding does not induce additional changes of DED conformation^22^. The NMR data and the current MD simulations both suggest that phosphorylation of C-terminal serine residues is enough to modulate the DED structure, converting the protein into a conformation that optimally binds to FADD.

The FADD structure exhibits significant changes upon PEA-15pp binding. The FADD DED in the complex does not show any significant changes from the free FADD (PDB ID 2GF5), with backbone RMSD between the bound and free forms of 2.118 Å. The death domain (DD) on FADD showed significant conformational change, involving the reorientation of helices α2, α3, and α4 relatively to the other three helices, and the backbone RMSD between the bound and free structures is 3.447 Å. The most remarkable change in FADD structure can be observed when the DEDs in the free and bound forms are superimposed. The DD in the bound structure is now rotated about 90° relative to the DED around the flexible linker between the two domains, moving closer to PEA-15pp (Fig. 4B,C). The relative reorientation between the two domains has significant implications in the DISC formation. An earlier crystal structure of human Fas and FADD DD assembly (PDB ID: 3EZQ) suggested that the two domains of FADD require a relative rotation to avoid steric clashing between the newly formed stem helix and C-helix on Fas DD and to expose the binding surface on the DED to recruit procaspase-8^26^. The crystal structure was limited by the absence of FADD DED in the construct. Our model provides the first direct evidence that FADD undergoes reorientation between the two domains, and the conformational change can be induced by either DD or DED interactions. Considering the oligomeric nature of the DISC, the relative reorientation of the DED and DD on FADD is necessary to expose its binding surfaces on both the DD and the DED. The reorientation between FADD DED and DD is likely dependent on the DED proteins recruited to the DISC, as PEA-15pp and procaspase-8 appear to bind to distinct surfaces on FADD DED (see “Discussion” below).

**Figure 5:**
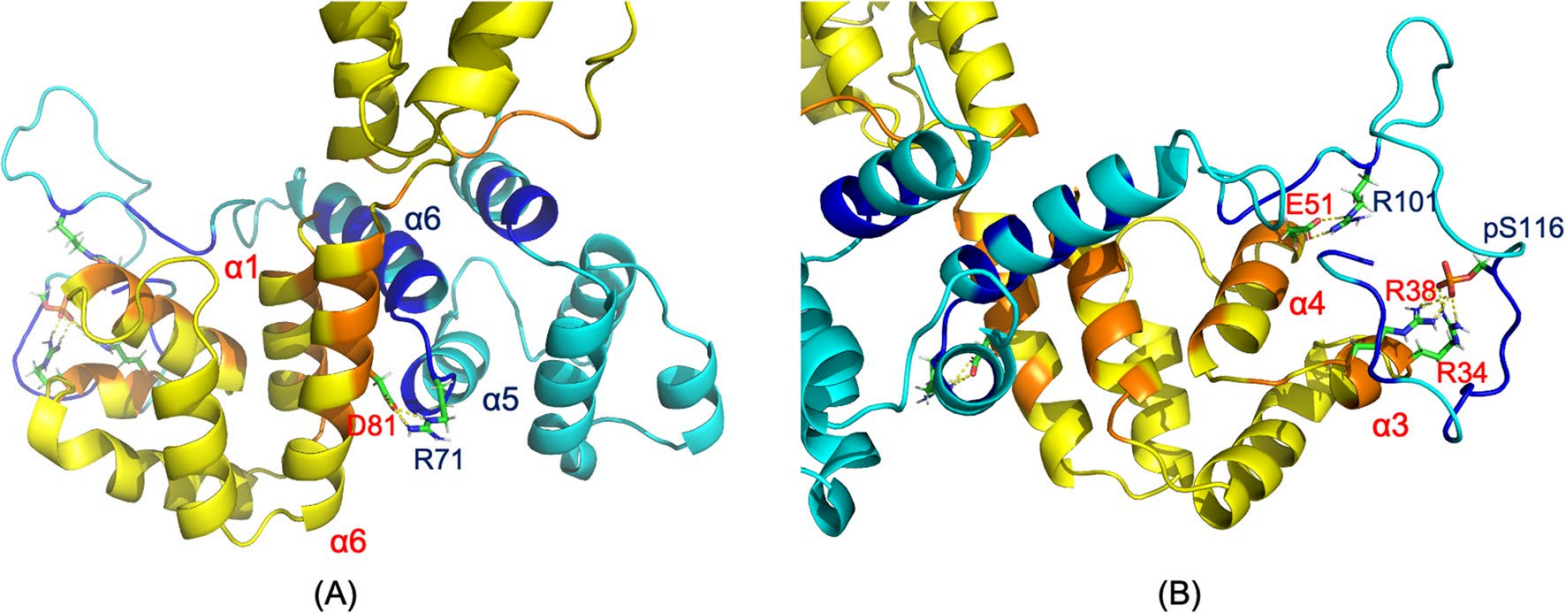
Binding interfaces in the simulated PEA-15pp/FADD complex. PEA-15pp is shown in cyan and FADD is shown in yellow. Interface residues on PEA-15 are colored in blue and residues on FADD are colored in orange. (**A**) DED-DED interface between PEA-15pp α5/α6 and FADD α1/α6, including direct interaction between PEA-15 R71 and FADD D81. (**B**) Interface between PEA-15pp C-terminal tail and FADD helices α3/α4, including direct interactions between PEA-15 R101 and FADD E51, and the strong interactions between PEA-15pp p-S116 and FADD R38 and R38. The molecular structures were visualized and generated using PyMOL version 2.4 (http://www.pymol.org/).

PEA-15pp and FADD engage in the canonical homotypic interaction within death superfamily proteins^27^, and the two proteins bind to each other using their respective DED surfaces. The binding surface on PEA-15pp is located at helices α5/α6 of the DED, which interacts with the α1/α6 surface of the FADD DED. The orientations of the two interacting DEDs are orthogonal to each other (Fig. 4B). Arg-71, located on the loop between helices α5 and α6 of PEA-15pp, is directly hydrogen-bonded to Asp-81, located on helix α6 of the FADD DED (Fig. 5A). It is worth noting that Arg-71 also engages in the interaction with ERK2 as discussed earlier. The C-terminal tail residues of PEA-15pp interact with residues on helices α3 and α4 of the FADD DED, including strong electrostatic and hydrogen-bonding interactions between *p*-Ser-116 on PEA-15pp and Arg-34 and Arg-38 on helix α3 of FADD DED, and the interaction between Arg-101 of PEA-15pp and Glu-51 on helix α4 of FADD DED (Fig. 5B). The atomic distances of these intermolecular interactions are plotted in Supplementary Fig. S6B. The Arg-71 of PEA-15 has relatively stable interaction with Asp-81 of FADD throughout the simulation, although there are some fluctuations at certain time points. The interactions between the phosphorylated Ser-116 of PEA-15 and the two arginine residues on FADD seemed to be stabilized early on at around 100 ns. The *p*-S116 interactions may have helped the formation of other intermolecular interactions involving the C-terminal tail residues, such as Arg-101, which only shows stable interaction with Glu-51 of FADD after around 140 ns. In the intermolecular regions of the pairwise mean-smallest-distance map for the PEA-15pp/FADD complex (Supplementary Fig. S7B), short distances can be observed in the PEA-15pp α5/α6 interaction with FADD DED α1/α6, as well as the PEA-15 C-terminal tail interaction with the α3/α4 of the FADD DED. Additionally, the FADD C-terminal tail interacts with PEA-15 α1 and α6. As expected, there are essentially no interactions between PEA-15 DED and FADD DD.

**Figure 6:**
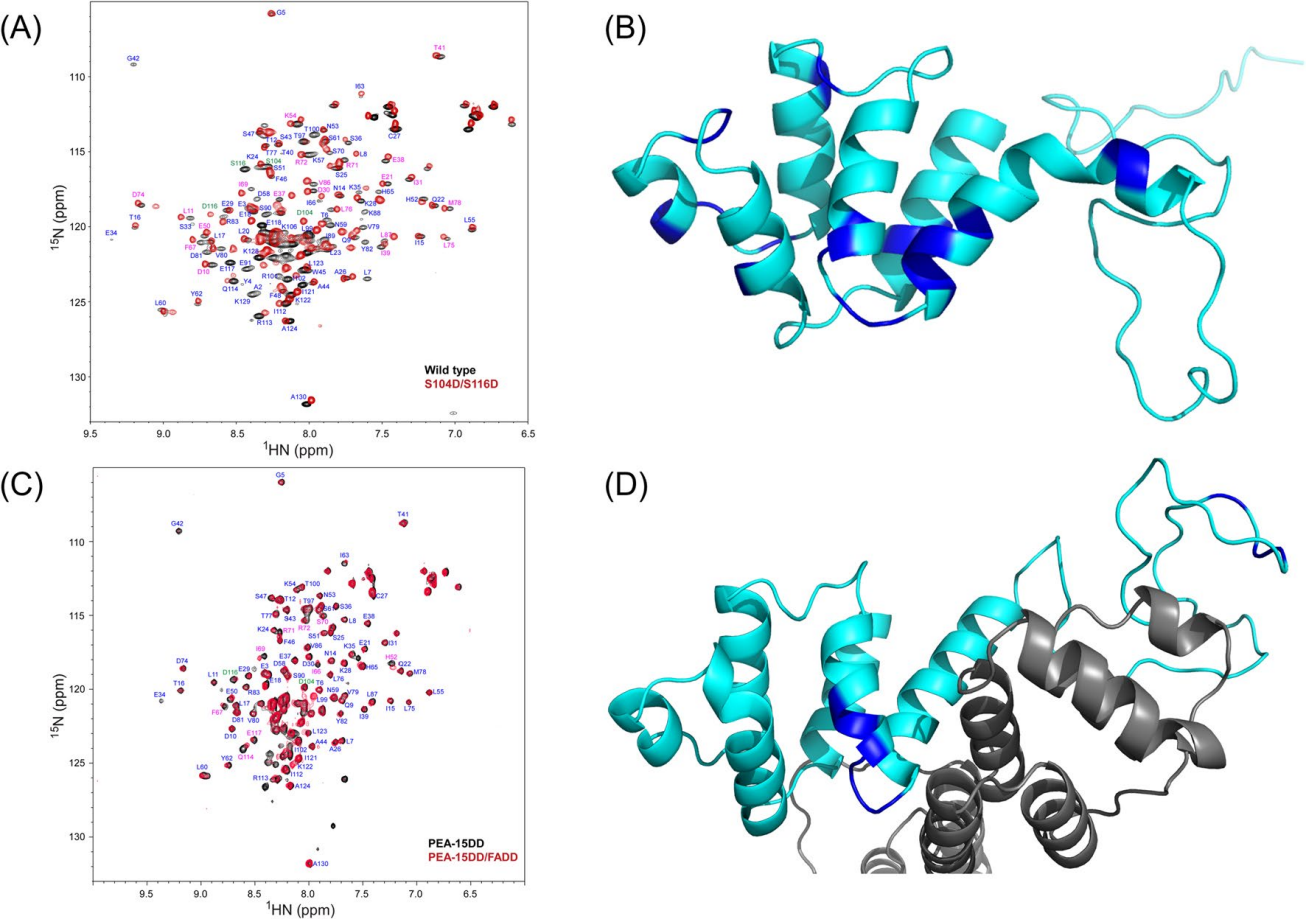
(**A**) Partial assignment of 2D HSQC ^1^H–^15^N correlation spectrum of PEA-15DD double mutant (red) overlaid with wild-type PEA-15 (black) spectrum. The assigned resonances are labeled next to the corresponding peaks. The resonances for wild-type S104 and S116 and the mutants S104D and S116D are labeled in green, the resonances experiencing significant chemical shift perturbation (CSP) upon mutation are labeled in magenta, and other resonances not shifted by mutation are labeled in blue. (**B**) Residues that experience significant CSP upon double mutation/double phosphorylation are mapped on the simulated PEA-15pp structure (cyan) and colored in blue. These residues include residues from helices α3, α5, and α6, as well as residues after the kink at the end of helix α6. (**C**) Partial assignment of 2D HSQC ^1^H–^15^N correlation spectra of PEA-15DD in its free form (black) and FADD-bound form (red). The available assignment for each resonance is labeled next to the peak. The two mutation sites, S104D and S116D, are labeled in green, the resonances experiencing significant CSP upon FADD binding are labeled in magenta, and other resonances not shifted by FADD binding are labeled in blue. (**D**) Residues that experience significant CSP on PEA-15DD (cyan) upon FADD binding are mapped on the simulated PEA-15pp/FADD structure and colored in blue. These residues are mostly from α5/α6 surface engaging in FADD binding. FADD is colored in grey. The NMR spectra in (**A**) and (**C**) were plotted using NMRViewJ version 9.2 (https://nmrfx.org/nmrfx/nmrviewj), and the molecular structures in (**B**) and (**D**) were visualized and generated using PyMOL version 2.4 (http://www.pymol.org/).

The simulated PEA-15pp/FADD model agrees well with our initial NMR assessment and partial backbone assignment of PEA-15 S104D/S116D double mutant (PEA-15DD), which has been shown to exhibit similar in vivo effects as the doubly phosphorylated PEA-15pp and to bind specifically to FADD^28^. The two-dimensional ^1^H-^15^N heteronuclear single-quantum coherence (HSQC) NMR spectrum of PEA-15DD displays significant chemical shift perturbations (CSPs) across a wide range of residues on the DED (Fig. 6A), indicating a significant conformational change upon double mutation, mimicking the doubly phosphorylated state. The residues experiencing large CSPs in the double mutant are mapped on the simulated PEA-15pp structure (Fig. 6B), including residues 37, 38, and 39 on helix α3, which display the most significant conformational change from the unphosphorylated state, residues 67–78 on helices α5/α6, which form the main binding surface to FADD, and residues 86 and 87, which are located on the kink of helix α6. In the NMR spectrum of the PEA-15DD/FADD complex, most residues do not experience additional CSP, indicating a largely stable conformation upon complex formation (Fig. 6C). The residues experiencing the most CSP between the free PEA-15DD and the FADD-bound form are mostly located on helices α5 and α6, including Arg-71, which engages in direct interaction with FADD. Other residues exhibiting significant shifts upon FADD binding include I66, F67, I69, S70, and R72 on the α5/α6 surface of the DED, and Q114, D116 (mutated from serine to mimic phosphorylation and involved in FADD binding), and E117 on the C-terminal tail. These residues are mapped on the simulated PEA-15pp/FADD complex structure (Fig. 6D).

### Surface polar interactions on PEA-15 and allosteric conformational modulation

The intrinsically disordered C-terminal tail of PEA-15 plays a determinant role in modulating the DED conformations. Upon binding to ERK DRS, the C-terminal tail allosterically modulates the DED conformation and exposes the α5/α6 surface to interact with the ERK2 DEF docking site. This allosteric modulation has been evidenced by our earlier NMR dynamic study^11^. Similarly, double phosphorylation on the C-terminal tail serine residues allosterically modulates the DED conformation and exposes the α5/α6 binding surface to FADD, ready the DED to accommodate the interacting partner. Upon FADD binding, a minimum conformational change of PEA-15pp is necessary. An analysis of the differential interactions on the PEA-15 surface at various phosphorylation and binding states affords the key to understanding the C-terminal tail-induced conformational changes at the DED.

On PEA-15, and most other DED-containing proteins, three charged amino acids, D19–R72–D74, form an electrostatic and hydrogen-bonded network, termed charge-triad. The distances between these three residues, however, are not consistent among different states of PEA-15 (Supplementary Fig. S8). The simulations show that for the free-form PEA-15, the D19–R72 interaction is more consistent despite small fluctuations over time, while the R72–D74 distance is in general quite large with no close contact. Upon phosphorylation at the C-terminal tail serine residues, the D19–R72 interaction is increasingly consistent with few fluctuations, and the R72–D74 distance closes with relatively small fluctuations. In the PEA-15/ERK2 complex, the R72–D74 interaction becomes more stable with very low fluctuations, while the D19–R72 interaction is destabilized. The same trend can be observed for PEA-15pp/FADD complex, in which the R72–D74 distance is very consistent with virtually no fluctuations, while the D19–R72 interaction becomes more fluctuated compared to the free-form PEA-15pp. Generally, the free-forms of PEA-15 favor the D19–R72 interaction, while the bound-forms favor the R72–D74 interaction. Phosphorylation at the PEA-15 C-terminal tail is sufficient to bring the R72–D74 distance closer, and FADD binding completely stabilizes the R72–D74 interaction.

In addition to the charge triad, there are numerous other polar interactions (i.e., electrostatic and hydrogen bonding) on the surface of PEA-15. The patterns of the polar interactions, however, vary depending on the phosphorylation status and the binding of protein partners. A list of these polar interactions on the PEA-15 surface is provided in Supplementary Table S1. The only non-charge-triad interaction consistently observed in all models is the one between Glu-21 and Lys-24. Other interactions identified in the DED of unphosphorylated free-form protein include Y4–E50, D30–Y62, K35–E38, E64–R83. Most of these interactions are broken when the conformational change is induced by phosphorylation, and new interactions are formed, including N14–R72, T16–D19, D30–K54, N59–S61. Upon binding to ERK2, many of the original interactions in the free-form are also broken, and a new interaction between D81–R85 is formed. The phosphorylated PEA-15pp has kept some of the same interactions in the FADD complex, indicating a less extensive conformational change switching from the free form to the bound form. Most of the shifts in polar interactions are at the binding interface formed by helices α5/α6. The changes of these polar interactions facilitate the C-terminal tail-induced conformational changes by reducing the transition energies between conformations.

### Phosphorylation-modulated binding specificity of PEA-15

Although it has been well-recognized that PEA-15 has a differential affinity to ERK1/2 and FADD in a phosphorylation status-dependent manner^13,19^, the underlying mechanism for the phosphorylation modulated preferential affinity has not been resolved. The comparison of the complex structures and interaction modalities between PEA-15/ERK2 and PEA-15pp/FADD specifically highlights the importance of the C-terminal phosphorylation in determining binding specificity.

**Figure 7:**
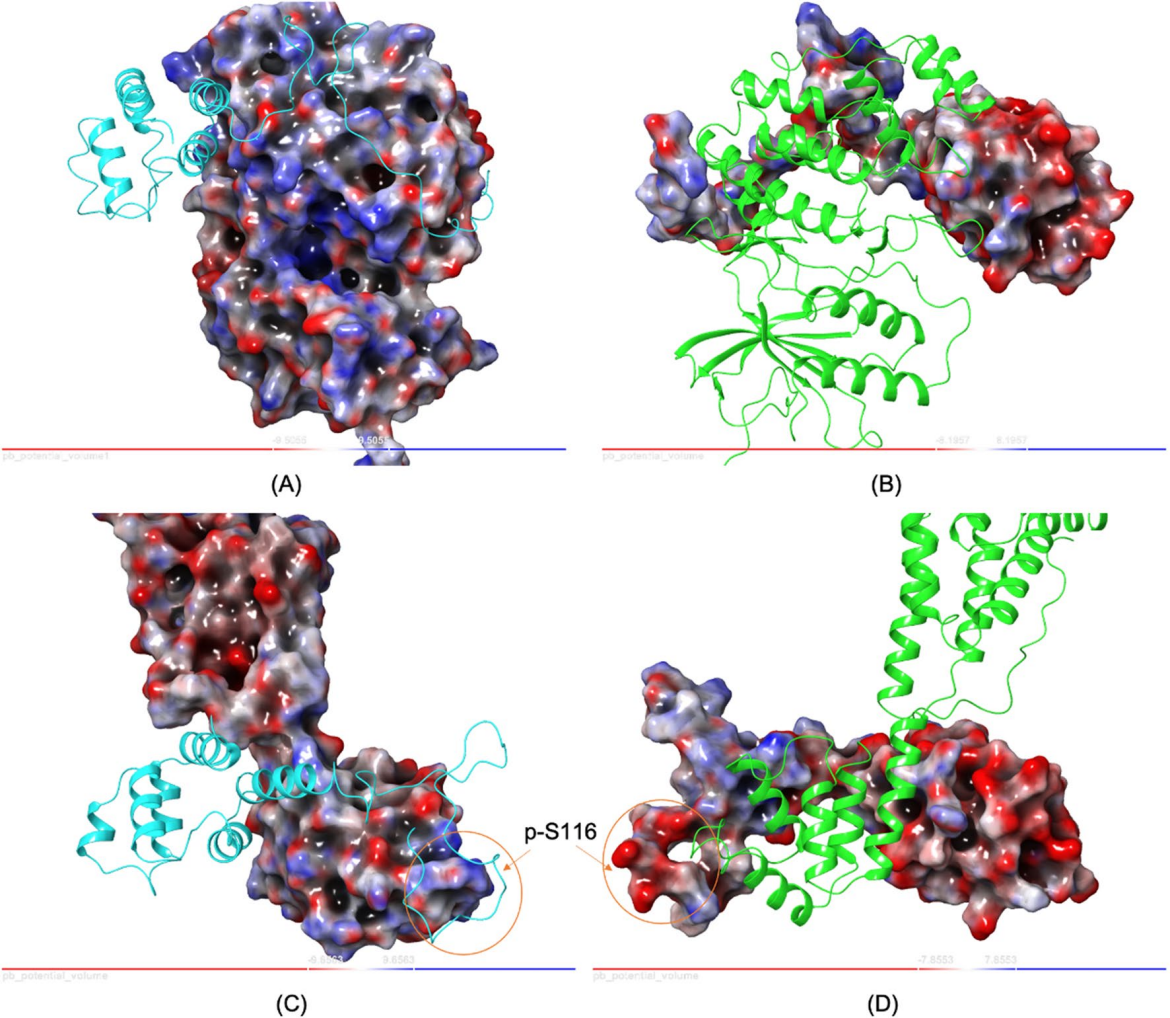
Poisson–Boltzmann Electrostatic Potential Surfaces (PB EPS) of PEA-15/ERK2 (**A**,**B**) and PEA-15pp/FADD (**C**,**D**) complexes. (**A**) ERK2 is shown as EPS and PEA-15 as a ribbon in cyan. (**B**) PEA-15 is shown as EPS and ERK2 as a ribbon in green. The view in (**B**) is rotated 180° from (**A**). (**C**) FADD is shown as EPS and PEA-15pp as a ribbon in cyan. (**D**) PEA-15pp is shown as EPS and FADD as a ribbon in green. The view in (**D**) is rotated 180° from (**C**). On the EPS, negative charge is represented as red and positive charge as blue. Protein–protein interactions are largely electrostatic in nature. Note that in PEA-15pp/FADD interaction, there is a strong electrostatic interaction between negatively charged p-S116 on PEA-15pp and the positive patch formed by R34 and R38 on FADD, which determines the binding specificity of PEA-15. The molecular structures and the EPS were generated using Maestro version 2021-2 (https://www.schrodinger.com/products/maestro).

The interactions between both PEA-15 and ERK2 and PEA-15pp and FADD are largely electrostatic, as shown on the electrostatic potential surfaces (EPS) of the complexes (Fig. 7). The PEA-15 surface on the helices α5/α6, used for interacting with both ERK2 and FADD, is primarily negatively charged, while both the ERK2 DEF binding site and FADD α1/α6 surface are mainly positively charged. The charge distribution and electrostatics at the PEA-15 C-terminal tail become more complicated and are dependent on phosphorylation. In the PEA-15/ERK2 interaction, PEA-15 utilizes its pseudo-d-peptide sequence at the end of the tail, which consists of several lysine residues and is highly positively charged, to interact with ERK2 DRS, which contains mostly negatively charged amino acids (Asp and Glu). In the PEA-15pp/FADD complex, the main interaction is the one between the highly negatively charged *p*-Ser-116, and the positive patch formed by two arginine residues on the helix α3 of the FADD DED. Another major intermolecular interaction in the PEA-15pp/FADD complex is formed between the negative charge on helix α1 of PEA-15pp and the positive charge on the FADD tail. The direct involvement of *p*-Ser-116 of PEA-15pp to interact with positively charged arginine residues on FADD plays a vital role in determining the phosphorylation-dependent binding specificity of PEA-15 in vivo. Unphosphorylated PEA-15, due to its neutral charge on Ser-116, cannot engage in strong electrostatic interaction with the positive arginine patch on FADD and tends to bind to ERK1/2. Phosphorylated PEA-15pp possesses negative charges on *p*-Ser-116 and switches the binding specificity to FADD through favorable electrostatic interaction.

## Discussion

As a versatile regulatory protein ubiquitously expressed in almost all mammalian cell types and tissues, PEA-15 controls multiple biological processes in a phosphorylation-dependent manner. The protein itself, however, does not seem to have any dedicated enzymatic or biological functions as PEA-15 knock-out (PEA-15^−/−^) did not appear to affect the health and fertility in mouse models^17^. As all functions of PEA-15 are exerted through engaging in protein–protein interactions (PPIs), it is intriguing to uncover how such a small, non-catalytic protein can modulate various cellular processes and bind structurally and functionally diverse proteins in the cell, all by modifying its phosphorylation states. In this study, we looked into the binding modes of PEA-15 with ERK2, a typical MAP kinase protein responsible for cell-cycle entry and cell proliferation^29^, and FADD, an adapter protein to facilitate the DISC assembly and promote the activation of initiator caspase-8 for extracellular death-ligand induced apoptosis^25^.

ERK2 and FADD do not share any sequence or structural homology, and they perform very distinct biological functions in cells. The cellular pathways they each involve do not seem to overlap. Structurally, ERK2 has a typical kinase fold, with a small N-lobe composed of a five-stranded antiparallel β-sheet containing the ATP-binding P-loop, and a large C-lobe with six conserved α-helical segments and the catalytic loop containing four short conserved β strands^30^. FADD is a death superfamily protein and is composed of an N-terminal DED and a C-terminal DD^25^, both having a six-helix bundled fold but differing in intermolecular interactions^27^. They both, however, bind to PEA-15 at proper phosphorylation states. When engaged in binding, PEA-15 utilizes both its DED and the C-terminal tail to interact with partner proteins through electrostatic interactions. The main binding surface on PEA-15 DED is formed by helices α5 and α6, which is negatively charged, while the surfaces of the ERK2 DEF docking site and FADD helices α1/α6 of its DED are both positively charged. PEA-15 uses the same binding surface on the DED to engage both ERK2 and FADD in the interactions. The in vivo binding specificity is determined by the phosphorylation states of the two serine residues on the PEA-15 C-terminal tail. When unphosphorylated, the C-terminal tail appears to be more positively charged due to the basic lysine residues towards the end of the tail. This positively charged tail will preferably interact with the negative patch of ERK2 DRS. When the C-terminal tail is phosphorylated, particularly at Ser-116, it dramatically changes the charge distribution on the tail, making it significantly more negatively charged. The change of the electrostatic property on the tail promotes the preferential binding to the positive patch on the FADD surface of DED helix α3. To our knowledge, this study is the first attempt to explain the phosphorylation-dependent binding specificity of PEA-15.

In this MD study, together with our previous NMR studies^11,22,31^, we pointed to a very flexible DED in PEA-15. PEA-15 DED adopts different conformations depending on its phosphorylation and binding status. The conformations are allosterically controlled by its intrinsically disordered C-terminal tail and mediated by surface polar interactions on the DED. When the C-terminal tail is recognized by ERK2 DRS, the DED modulates its conformation, including the allosteric exposure of α5/α6 binding surface and relaxation of α3 to facilitate its interaction with the DEF docking site on ERK2. Similarly, when the C-terminal tail serine residues are phosphorylated, it allosterically modulates DED conformations to prepare it to engage in DED–DED interactions. When it comes to bind with FADD, little conformational change is needed to form the complex.

On almost all DED-containing proteins, a prominent surface feature, termed charge-triad, consisting of three charged residues, D/E-RxDL, in which the first charged residue can be either D or E, and x indicates any amino acids, can be identified to form a hydrogen-bonded and electrostatic network^27^. For PEA-15, we have illustrated the charge-triad network, D19-R72-D74 in our high-definition NMR structure^9^. However, the role of the conserved charge-triad has not been determined. Although it was stipulated that D74 is involved directly in ERK2-binding, as D74A mutation caused the loss of binding capacity of PEA-15 to ERK2^8^, later crystal structures^21^ and NMR dynamics study^11^ both rejected this hypothesis. We proposed that the charge-triad, through the hydrogen-bonded network, facilitate the conformational change at the DED by reducing transition energy between free- and bound-conformations. The current MD simulations confirmed our hypothesis. In the free-form PEA-15, D19 is hydrogen-bonded to R72, while in the ERK2-bound form, R72 is hydrogen-bonded to D74, making the switch between free- and bound-conformations swiftly. For the PEA-15pp/FADD complex, the same trend seems to hold. D19-R72 interaction dominates in the free PEA-15pp, while R72-D74 interaction is more prevalent in the bound form. D74A mutation will disrupt the interaction between R72 and D74, which is essential in mediating complex formation. As we pointed out earlier, other surface polar interactions on PEA-15 also contribute to the conformational transition by lowering the transition energies.

**Figure 8:**
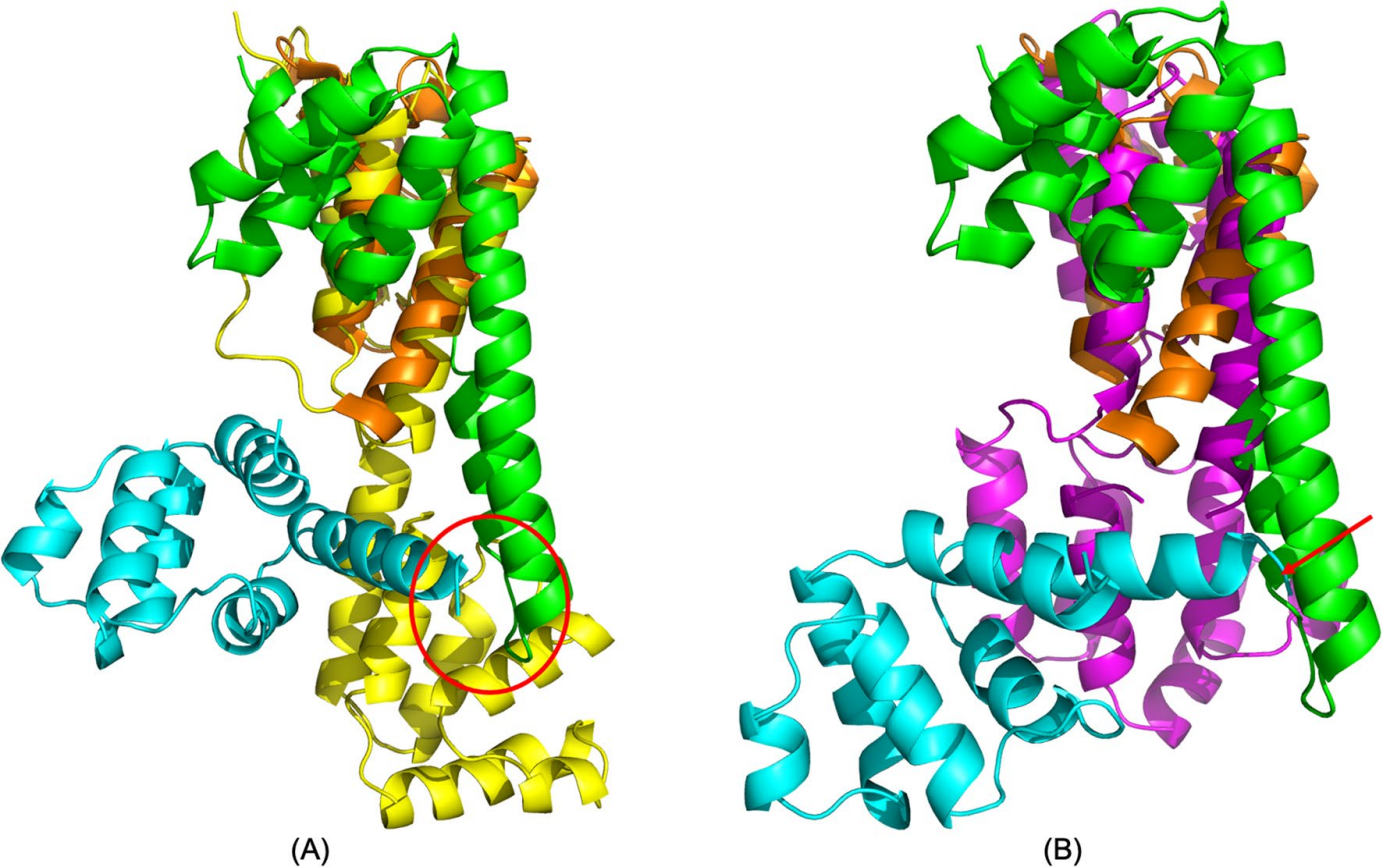
(**A**) Overlay of simulated PEA-15pp/FADD complex (PEA-15pp in cyan and FADD in yellow) onto Fas–FADD DD complex crystal structure (Fas DD in green and FADD DD in orange), 3EQZ, over FADD DD residues. The simulated FADD DD structure matches very well with the crystal structure with the RMSD of 1.610 Å. (**B**) Overlay of NMR structure of intact FADD (magenta), 2GF5, onto Fas–FDD DD complex crystal structure (Fas DD in green and FADD DD in orange), 3EQZ over FADD DD residues. The RMSD between FADD DD is 4.334 Å, indicating a conformational change in the DD upon complex formation. Assuming PEA-15pp binds to the same position of FADD DED (shown in cyan), its helix α6 will clash with the newly formed stem helix and C-helix on Fas DD (indicated by red arrow). In our simulated structure, however, due to the relative repositioning of FADD DD and DED, the bound PEA-15 DED can avoid such steric hindrance, indicated as the red circle in (**A**). The C-terminal tail of PEA-15pp is not shown for clarity. The molecular structures were visualized and generated using PyMOL version 2.4 (http://www.pymol.org/).

Another significant finding in this study is that FADD undergoes a large conformational change upon interaction with PEA-15pp, inducing a complete reorientation of the DD relative to the DED. This reorientation between the two domains has significant implications in the DISC formation in responding to extracellular apoptotic signals. Although there are currently no complete structural models for the DISC, two crystal structures have been reported for the complexes formed between the DD of the death receptor Fas, and the DD of the adaptor protein FADD, with PDB IDs 3EZQ^26^ and 3OQ9^32^. The asymmetric oligomeric DD assembly formed between mouse Fas and human FADD DDs (3OQ9) revealed three different types of binding interfaces in the association of DD complexes, reflecting the complexity of maintaining an intricate balance between the regulatory threshold and responsive sensitivity in triggering apoptosis^32^. The tetrameric human Fas–FADD DD complex displayed intriguing new features, in which an elongated new stem helix is formed in place of helices α5 and α6, and an additional C-helix is formed in the C-terminus of Fas DD^26^. The stem helix and the C-helix may promote the association of Fas and the formation of a regulatory bridge between Fas molecules. Another effect of shifting helix α6 and fusing with helix α5 to form the stem helix is to expose a hydrophobic patch between helices α1 and α5 of Fas DD to interact with helices α1 and α6 of FADD DD. This crystal structure implied that relative repositioning of the two domains of FADD was deemed to be necessary to expose procaspase-8 binding surface and to avoid the steric clash with the newly formed stem helix and C-helix of Fas DD. The NMR structure of full-length FADD (2GF5) showed that the Fas binding surface on DD (helices α1/α6) and the PEA-15pp binding surface on DED (α1/α6) are located on the same side of the protein. Due to the oligomeric nature of the DISC, it seems only possible to bind to both Fas DD and another DED if the two domains on FADD undergo a reorientation, exposing surfaces on both DD and DED. Our MD simulation of the PEA-15pp/FADD complex provides the initial evidence of the repositioning of FADD DED relative to DD, shifting the binding surface at helices α1/α6 for DED–DED interaction, and avoiding clashing with the stem helix and C-helix on Fas DD. The FADD DD conformation in our simulated complex structure agrees very well with the crystal structure 3EZQ, with a backbone RMSD of 1.610 Å. The rotation of DED relative to DD makes the bound PEA-15 DED, particularly helix α6, to avoid the steric hindrance with the stem helix or the C-helix of Fas DD (Fig. 8A). As a comparison, the NMR structure of full-length FADD (2GF5) had a backbone RMSD of 4.334 Å for the DD with crystal structure 3EZQ, and if we assume PEA-15 binding to the same surface at helices α1/α6 of FADD DED, significant clashing between PEA-15 helix α6 and the stem helix and C-helix is unavoidable (Fig. 8B). The relative reorientation between the two domains of FADD appears to be vital in the DISC formation and procaspase-8 activation.

Due to the severe consequence of apoptosis, the process is tightly regulated. In addition to PEA-15, cellular Fas-associated death domain-like interleukin-1β-converting enzyme-inhibitory protein (c-FLIP), another DED-containing protein, acts as an endogenous antiapoptotic protein, blocking the activation of procaspase-8 and -10^33^. Through alternative splicing, three isoforms of c-FLIPs have been identified: the long form (c-FLIP_L_), the short form (c-FLIP_s_), and the Raji form (c-FLIP_R_)^34^. Both the long and short forms of c-FLIP can be recruited to the DISC, where the long form is processed in a similar manner of activation of procaspase-8^35^. Although both doubly phosphorylated PEA-15 and c-FLIPs are capable of regulating apoptosis mediated by the tumor necrosis factor α (TNFα) receptor family, they differ in terms of structures, inhibition mechanisms, and expression levels in different cell and tissue types. c-FLIPs are consist of tandem DEDs, similar to those of procaspase-8 and -10^36^, while PEA-15 has only one DED, and its antiapoptotic activity is phosphorylation-state dependent^13,19^. Differential expressions of PEA-15 and c-FLIPs have been observed in various cell types. In human malignant glioma cell lines, PEA-15 mRNA is expressed two-fold higher levels in tumor necrosis factor-related apoptosis-inducing ligand (TRAIL)-resistant cell lines compared to sensitive cells, while c-FLIP mRNA expression is similar in both sensitive and resistant cells^18^. In CD95-resistant thyroid tumor cells, both PEA-15 and c-FLIP mRNA expressions are up-regulated through interleukin-4 (IL-4) and IL-10^37^. In terms of inhibition mechanism and binding interface, earlier protein–protein docking models suggested that c-FLIP preferentially uses its α2 helix on DED2 to interact with α1/α4 surface on the DED of FADD, and procaspase-8 uses its α1 helix on DED1 to interact with the α2/α5 surface on FADD DED^38^. In our model, PEA-15pp engages in the interaction with its α5/α6 surface to bind to the α1/α6 surface, and with its C-terminal tail to bind to the α3/α4 surface on FADD DED. The interactions between c-FLIP/FADD and procaspase-8/FADD are predominantly hydrophobic, while the PEA-15DD/FADD interaction is largely electrostatic. The FADD DED α2/α5 binding surface to procaspase-8 is not directly blocked by either PEA-15pp or c-FLIP tandem DEDs. However, the relative reorientation of FADD DED and DD may hide the α2/α5 DED surface into the oligomeric assembly of the DISC, making it inaccessible to recruit procaspase-8. As procaspase-8 and apoptosis inhibitors, including PEA-15pp and c-FLIPs, bind to distinct surfaces on FADD DED, the specific reorientation between FADD DD and DED would be dependent on the proteins to be recruited to the DISC, and expose respective binding surface on the DED. The availability of both c-FLIPs and PEA-15 as endogenous apoptosis regulators provides additional control mechanisms to fine-tune the quantity and dynamics of pro- and anti-apoptotic pathways. As PEA-15 is abundantly expressed in almost all types of cells, we speculate that PEA-15 may act as a first responder to control and balance these pathways through relatively quick phosphorylation and dephosphorylation processes.

## Methods

### Construction of initial free-form and complex models

The PEA-15 in its free-form was constructed from the NMR structure, PDB ID 2LS7^9^, by adding the C-terminal tails residues 91–130 as random coil using the comparative protein structure modeling program Modeller^39–42^. The PEA-15pp model was constructed from the final simulated structure of the unmodified PEA-15 by adding phosphoryl group to each of Ser-104 and Ser-116 residue using the posttranslational modification plugin, PyTMS^43^, for the molecular graphics system PyMOL (Schrödinger, LLC., New York, NY).

The complex model between PEA-15 and ERK2 was constructed from the crystal structure of the complex, PDB ID 4IZ5^21^. The missing residues in the PEA-15 chain of the crystal structure were rebuilt from the Rosetta model of ERK2-bound PEA-15, PDB ID 6P6C^22^, using the Modeller program. The T185E mutation in the ERK2 of the crystal structure was also changed back to a threonine using Modeller.

As the complex structure between PEA-15 and FADD is unavailable, the complex model between PEA-15pp and FADD was constructed using the final simulated PEA-15pp structure docked with the NMR structure of intact FADD, PDB ID 2GF5^25^, using online SwarmDock Server^44–46^. Among the 10 best docking models generated by the Server, the model selected for further MD simulations matches best with the NMR chemical shift perturbation data, which is indicative of the protein–protein binding interface between the two proteins (Fig. 6C).

### MD simulation protocols

All-atom MD simulations with explicit solvent were performed with the parallel molecular dynamics package GROMACS^47–53^ with CUDA-based GPU acceleration on an Exxact workstation equipped with two Nvidia Tesla V100 GPUs running CentOS 8 and GROMACS version 2020-3. The simulation timescale was 50 ns for both the full-length, unmodified PEA-15 model and the doubly phosphorylated PEA-15pp model, and 150 ns for the two complexes of PEA-15/ERK and PEA-15pp/FADD. All simulations were conducted at neutral pH, 300 K, and 1 atm. CHARMM36 force field^54^ was utilized for all protein parameters.

All starting protein structures were solvated in a cubic box with boundaries extended at least 1.0 nm in all directions from the protein molecules, and filled with TIP3P water molecules and an appropriate number of counter ions (either Na^+^ or Cl^−^) to neutralize the total charge of the system. Energy minimization was performed using the steepest descent algorithm with convergence on *F*_max_ < 1000 kJ mol^−1^ nm^−1^. Equilibration was conducted in two-phased, 100 ps under *NVT* (constant number of particles, volume, and temperature) and 100 ps under *NPT* (constant number of particles, pressure, and temperature) ensembles, while restraining atomic position. The coupling time constant was set to 0.1 ps for the equilibration, and periodic boundary conditions were applied with a constant temperature of 300 K and the constant pressure of 1 atm. Heavy atom bond lengths were constrained using Linear Constraint Solver (LINCS) algorithm. For the production MD run, both Coulomb and van der Waals interactions were truncated at 1.2 nm, with Particle Mesh Ewald (PME) summation method for the long-range electrostatic interactions. Production runs (50 ns for free-form proteins and 150 ns for protein complexes with a timestep of 2 fs) were performed upon the equilibrated system using CHARMM36 force field and the leap-frog algorithm utilizing both NVidia Tesla V100 GPUs for acceleration. Snapshots of conformations were collected at every 100 ps.

### Analysis of MD results

MD trajectories were analyzed using GROMACS tools. Secondary structural content was determined using program DSSP^55^. Root-mean-square deviation (RMSD) from the starting structure and root-mean-square fluctuation (RMSF) to highlight highly flexible regions were calculated using the Cartesian coordinates of the Cα atoms. The RMSD for free-form PEA-15 proteins stabilized at around 30 ns due to the flexible C-terminal tail, and the final 20-ns trajectories were utilized for the analysis. The RMSD for the complex structures stabilized at around 100 ns, and the last 50-ns trajectories were included in the analysis. Various intramolecular and intermolecular hydrogen bonding distances were calculated to map out the polar surface interaction network on PEA-15 and the intermolecular interactions that stabilize the complex structures.

### Calculation of electrostatic potential surfaces

The EPS of each individual protein in the PEA-15/ERK2 and PEA-15pp/FADD complexes were calculated with the APBS (Adaptive Poisson-Boltzmann Solver) method^56^ implemented in the Maestro program (Schrödinger, LLC, New York, NY). This method creates a molecular surface and colors the Poisson–Boltzmann (PB) potential onto the protein surface. The negative potential is colored by red and the positive potential is colored by blue. A solvent dielectric constant of 80 and a solvent radius of 1.4 Å were used in the calculations.

### Molecular visualization

All molecular models were visualized and produced using either PyMOL or Maestro, including structural superposition and comparison, distance and hydrogen-bonding analysis, protein–protein interface analysis, and EPS analysis.

## Supplementary Information

Supplementary Information.
