# Supplementary material for: PEA-15 engages in allosteric interactions using a common scaffold in a phosphorylation-dependent manner
Source: Sci Rep. 2022 Jan 7;12:116. doi: 10.1038/s41598-021-04099-6 (PMC8742051; doi:10.1038/s41598-021-04099-6)
Supplement: Supplementary file 1 — Supplementary Information. [file 41598_2021_4099_MOESM1_ESM.pdf]

PEA-15 Engages in Allosteric Interactions Using a Common Scaffold in a Phosphorylation-Dependent Manner

Joyce Ikedife[1], Jianlin He[2], Yufeng Wei[1,*]

1. Department of Chemistry, New Jersey City University, Jersey City, NJ 07305, USA

2. Third Institute of Oceanography, Ministry of Natural Resources, Xiamen, Fujian 361005, CHINA

Supplementary Table

Table S1. PEA-15 intramolecular surface polar contacts

PEA-15	PEA-15pp	PEA-15/ERK2	PEA-15pp/FADD
	E3/OE2 – R85/NH2<br>0.292 ± 0.034		
	E3/OE1 – K106/NZ<br>0.273 ± 0.009		
Y4/OH – E50/OE1<br>0.298 ± 0.063			
	N14/OD1 – R72/NH1<br>0.277 ± 0.010		
	T16/OG1 – D19/OD2<br>0.270 ± 0.014		
**D19/OD2 – R72/NH2**<br>0.305 ± 0.037	**D19/OD2 – R72/NH2**<br>0.279 ± 0.013	**D19/OD2 – R72/NH2**<br>0.295 ± 0.042	**D19/OD2 – R72/NH2**<br>0.310 ± 0.052
E21/OE1 – K24/NZ<br>0.286 ± 0.055	*E21/OE2 – K24/NZ*<br>0.298 ±0.084	*E21/OE2 – K24/NZ*<br>0.300 ± 0.089	*E21/OE2 – K24/NZ*<br>0.317 ± 0.097
D30/OD2 – Y62/OH<br>0.308 ± 0.069	D30/OD2 – K54/NZ<br>0.300 ± 0.062		D30/OD2 – K54/NZ<br>0.324 ± 0.097
K35/NZ – E38/OE1<br>0.284 ± 0.069			K35/NZ – E38/OE1<br>0.280 ± 0.052

	N59/OD1 – S61/OG<br>0.278 ± 0.033		
E64/OE1 – R83/NH1<br>0.281 ± 0.012			
E64/OE2 – K106/NZ<br>0.308 ± 0.078			
E68/OE2 – K106/NZ<br>0.344 ± 0.140			
	**R72/NH1 – D74/OD2**<br>0.301 ± 0.047	**R72/NH1 – D74/OD2**<br>0.279 ± 0.011	**R72/NH1 – D74/OD2**<br>0.282 ± 0.013
	D81/OD1 – K107/NZ<br>0.299 ± 0.059	D81/OD1 – R85/NH2<br>0.301 ± 0.060	
	D93/OD1 – K109/NZ<br>0.270 ± 0.018		
			pS104/O2P – K106/NZ<br>0.270 ± 0.016
	R113/NH2 – pS116/O2P<br>0.278 ± 0.013		

Each entry consists of the interacting atom pairs within PEA-15 protein, and its corresponding distance (mean ± standard deviation in nm). The charge-triad interactions are listed in **boldface**. E21-K24 interaction is the only other polar interaction consistently observed in all models, and is shown as *italics*.

Supplementary Figures

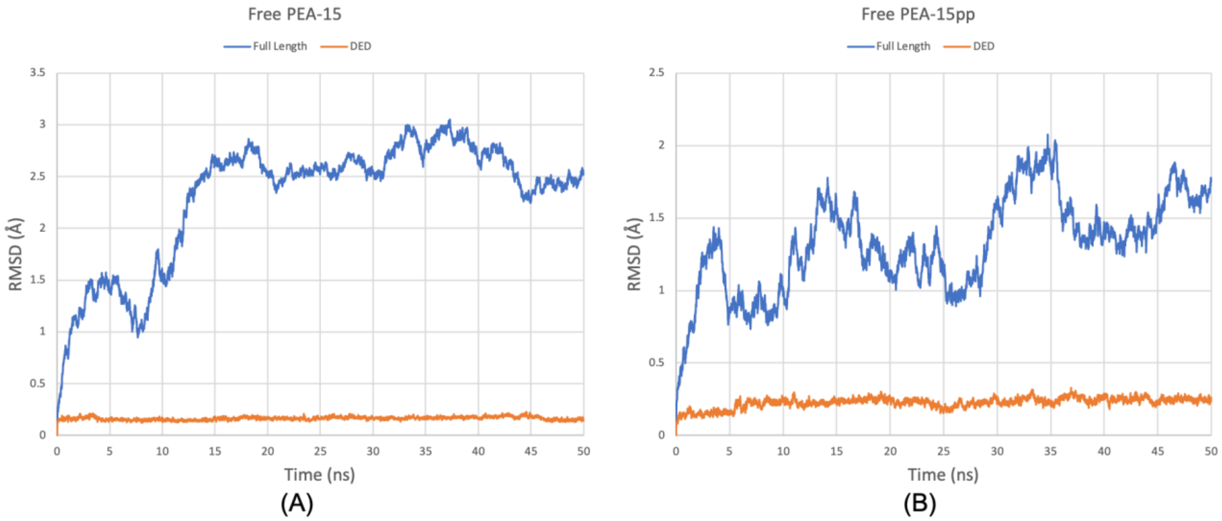

Figure S1. RMSD for 50-ns MD simulations of (A) unphosphorylated PEA-15 and (B) phosphorylated PEA-15pp. The full-length RMSD values for both proteins are much higher than the corresponding DED (residues 1-90) only due to the highly flexible C-terminal tail (residues 90-130), which can adopt a wide variety of conformations. The DED RMSD indicated that the simulations went to equilibrium at about 30 ns.

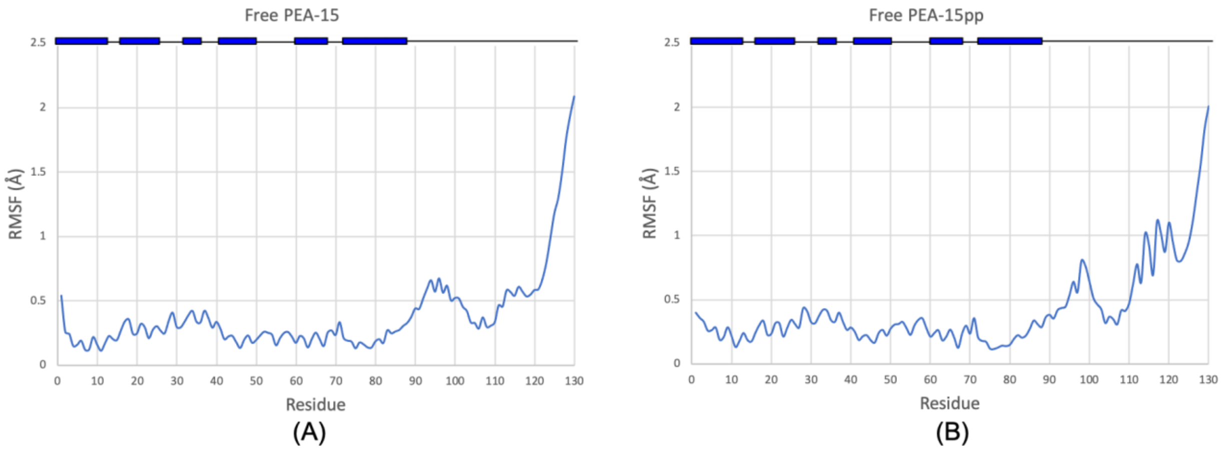

Figure S2. Per-residue RMSF of (A) unphosphorylated PEA-15 and (B) phosphorylated PEA-15pp. The blue bars on top of each graph indicate the positions of the six helices of the DED. For both proteins, the folded DED has relatively low RMSF, while the C-terminal residues have much higher RMSF values, indicating the flexibility of the tail. The blue bars on top of each graph indicate the relative position of the six helices of the PEA-15 DED.

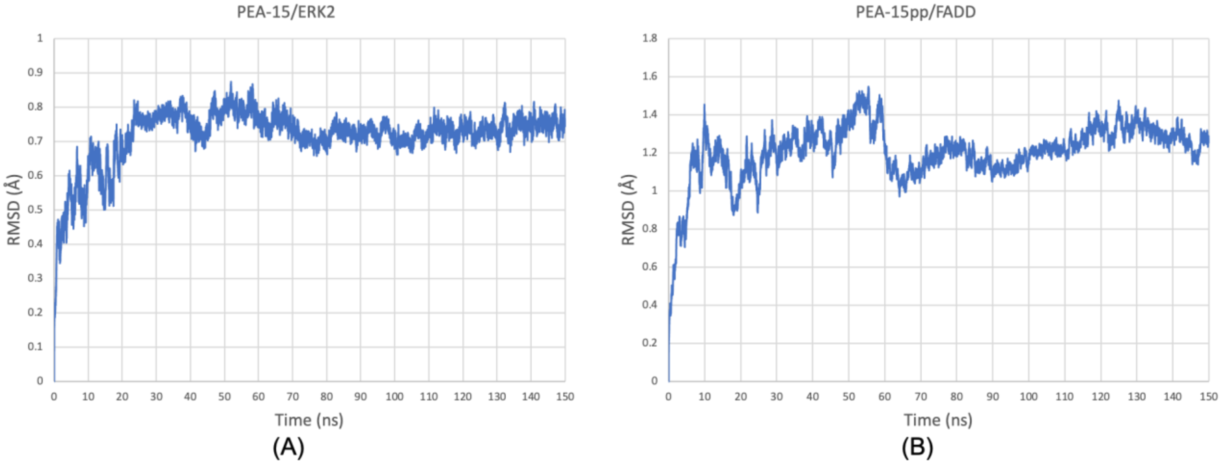

Figure S3. RMSD for 150-ns MD simulations of (A) unphosphorylated PEA-15/ERK2 and (B) phosphorylated PEA-15pp/FADD complexes. Both complex simulations stabilized at around 100 ns. The variations in the RMSD, particularly in the PEA-15pp/FADD complex, are due to the flexibility, although greatly reduced comparing to their free forms, of the C-terminal tail.

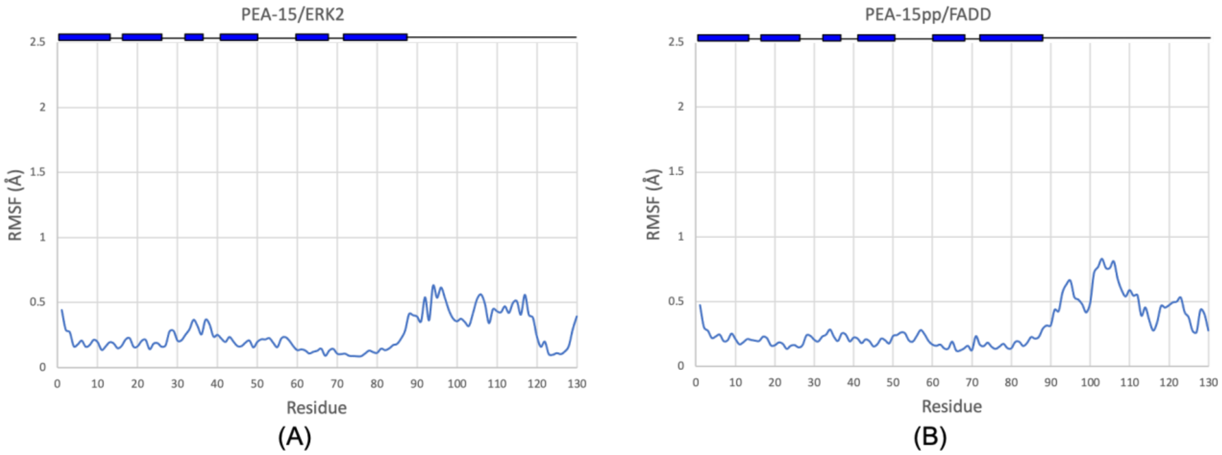

Figure S4. Per-residue RMSF of (A) unphosphorylated PEA-15/ERK2 and (B) phosphorylated PEA-15pp/FADD complexes. The blue bars on top of each graph indicate the positions of the six helices of the DED. For both complexes, the folded DED has relatively low RMSF, comparable to the free-form proteins. Although the RMSF values for C-terminal residues are still higher than those of the DED, the values are now much lower comparing to the tail RMSF in free-form proteins, indicating that strong binding to their respective partners significantly restricted the motion and flexibility of the tail. The blue bars on top of each graph indicate the relative position of the six helices of the PEA-15 DED.

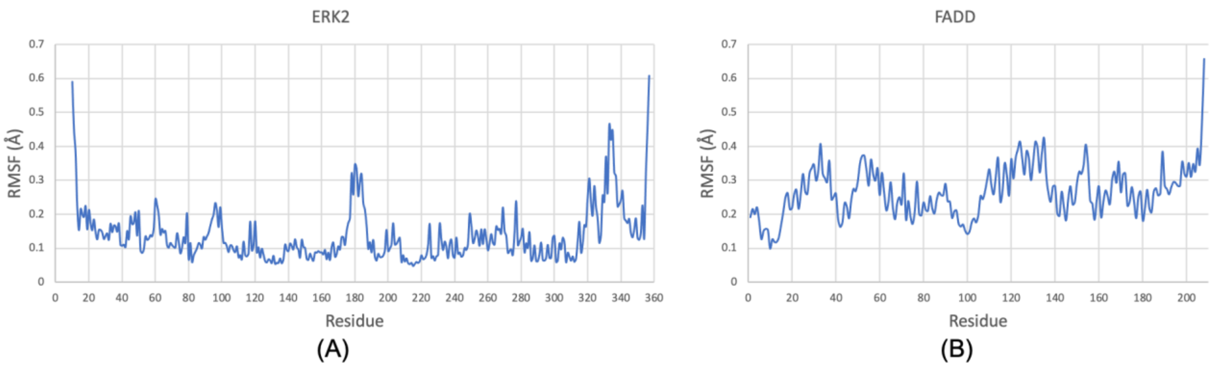

Figure S5. Per-residue RMSF of (A) ERK2 in PEA-15/ERK2 complex and (B) FADD in PEA-15pp/FADD complex. (A) The ERK2 structure in the complex did not display much difference from the reported crystal structure, and the fluctuation is relatively low for regions with regular secondary structures. (B) FADD is relatively flexible in the PEA-15pp complex. The conformation of FADD exhibits some significant changes from the free-form protein.

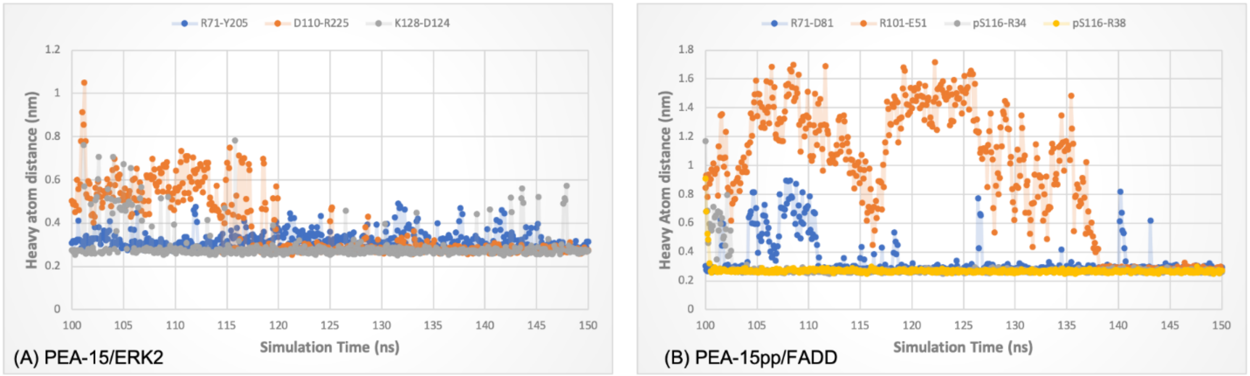

Figure S6. Heavy atom distances at the interfaces over time between (A) PEA-15 and ERK2, including PEA-15 R71 and ERK2 Y205 (blue), PEA-15 D110 and ERK2 R225 (orange), and PEA-15 K128 and ERK2 D124 (grey); and (B) PEA-15pp and FADD, including PEA-15pp R71 and FADD D81 (blue), PEA-15pp R101 and FADD E51 (orange), and PEA-15 p-S116 and FADD R34 (grey) and R38 (yellow). The first residues shown in the legends are from PEA-15, and the second residues are from ERK2 (A) or FADD (B). The interactions between p-S116 and the two arginines are FADD are particularly strong, which stabilizes other interactions between PEA-15pp C-terminal tail and FADD. The strong interactions between p-S116 with positively charged FADD residues provides mechanistic insight on phosphorylation-dependent binding specificity.

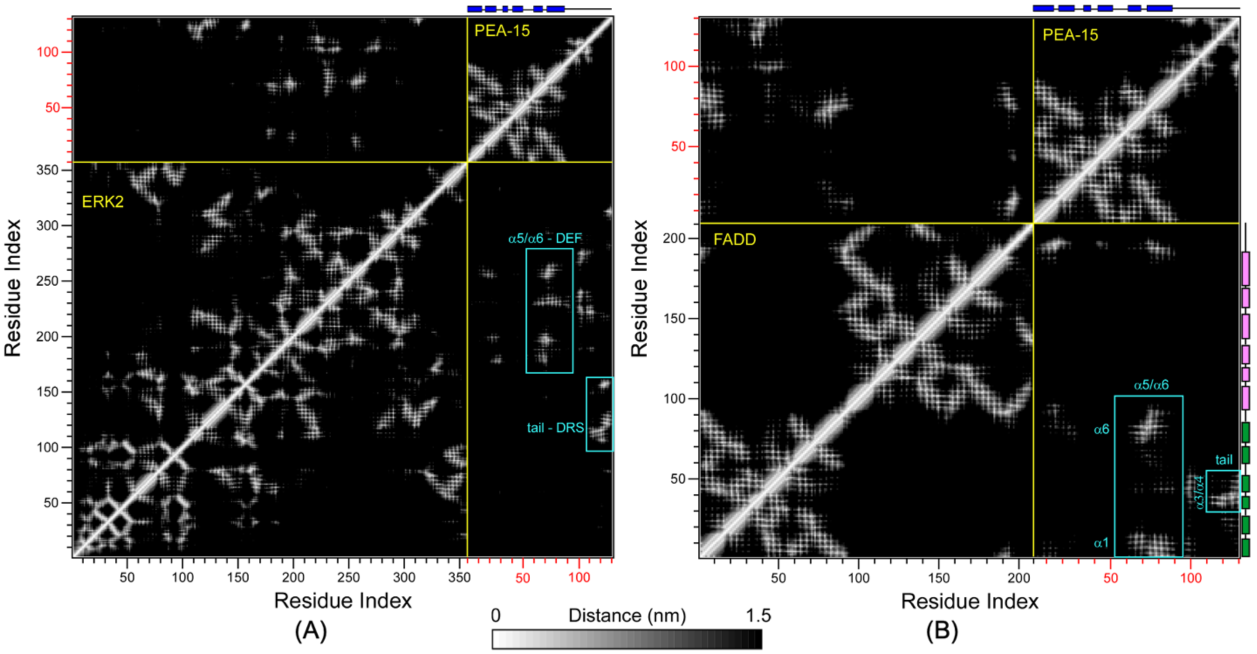

Figure S7. Pairwise mean-smallest-distance maps for (A) PEA-15/ERK2 complex, and (B) PEA-15pp/FADD complex. The maps show specific intermolecular interactions in the complexes as indicated as cyan boxes. Blue bars on top of PEA-15 sequence indicate the positions of the six helices of PEA-15 DED, and green and magenta bars to the right of FADD sequence indicate the positions of six helices of DED and DD, respectively.

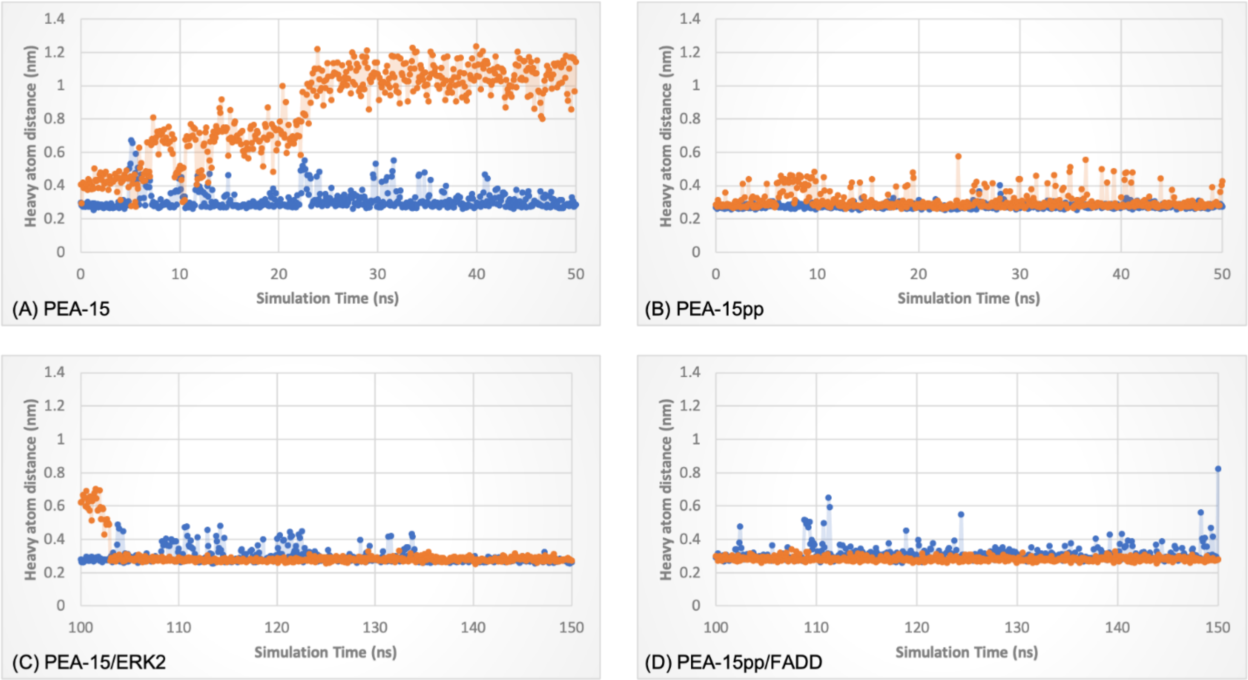

Figure S8. Heavy atom distances in charge-triad, D19-R72-D74, of (A) free-form PEA-15, unphosphorylated, (B) free-form PEA-15pp, doubly phosphorylated, (C) PEA-15/ERK2 complex, and (D) PEA-15pp/FADD complex. D19-R72 distance is colored in blue, and R72-D74 distance is colored in orange. In the free forms (A and B), D19-R72 is more likely to engage in hydrogen bond, while R72-D74 shows more fluctuations. In the complex structures (C and D), D19-R72 displays more fluctuation, while R72-D74 is more engaged in hydrogen bond with essentially no fluctuations.
